# Memetic Differential Evolution with an Improved Contraction Criterion

**DOI:** 10.1155/2017/1395025

**Published:** 2017-04-04

**Authors:** Lei Peng, Yanyun Zhang, Guangming Dai, Maocai Wang

**Affiliations:** ^1^School of Computer Science, China University of Geosciences, No. 388 Lumo Road, Hongshan District, Wuhan, China; ^2^Hubei Key Laboratory of Intelligent Geo-Information Processing, China University of Geosciences, Wuhan 430074, China

## Abstract

Memetic algorithms with an appropriate trade-off between the exploration and exploitation can obtain very good results in continuous optimization. In this paper, we present an improved memetic differential evolution algorithm for solving global optimization problems. The proposed approach, called memetic DE (MDE), hybridizes differential evolution (DE) with a local search (LS) operator and periodic reinitialization to balance the exploration and exploitation. A new contraction criterion, which is based on the improved maximum distance in objective space, is proposed to decide when the local search starts. The proposed algorithm is compared with six well-known evolutionary algorithms on twenty-one benchmark functions, and the experimental results are analyzed with two kinds of nonparametric statistical tests. Moreover, sensitivity analyses for parameters in MDE are also made. Experimental results have demonstrated the competitive performance of the proposed method with respect to the six compared algorithms.

## 1. Introduction

In 1989, the name of “memetic algorithms” (MAs) [1] was introduced for the first time. In the last two decades, MAs gradually became one of the recent growing areas of research in evolutionary computation. They combine various evolutionary algorithms (EAs) with different LS methods to balance exploration and exploitation. Existing examples of memetic algorithms are NM-BRO [2], MA-LSCh-CMA [3], LBBO [4], IMMA [5], and MPSO [6]. In the framework of MAs, LS operators are used to execute further exploitation for the individuals generated by common EA operations, which is helpful to enhance the EA's capacity of solving complicated problems.

Differential evolution was first proposed by Storn and Price [7] in 1995 to solve global numerical optimization problems over continuous search spaces. It shares some similarities with other EAs. For example, DE works with a population of solutions, called vectors; it uses recombination and mutation operators to generate new vectors and, finally, it has a replacement process to discard the less fit vectors. DE represents solutions with real coding. Some of the differences with respect to other EAs are as follows: DE uses a special mutation operator based on the linear combination of three individuals and uses a uniform crossover operator. It has several attractive features. DE is relatively simple to implement and was demonstrated to be very effective on a large number of cases. In the past few decades, DE has been successfully used in many real-world applications, such as space trajectory design [8–10], hydrothermal optimization [11], underwater glider path planning [12], and vehicle routing problem [13].

Despite its successful applications to different classes of problems in different fields, DE was demonstrated to converge to a fixed point, a level set [10], or a hyperplane not containing the global optimum [14]. Furthermore, in some cases it was shown to have slow local convergence.

In order to overcome these shortcomings, some authors have proposed a hybridization of DE with some local search heuristics. dos Santos Coelho and Mariani [15] proposed a version of memetic DE which combines DE with the generator of chaos sequences and sequential quadratic programming technique (DEC-SQP). In this memetic algorithm, DE with chaos sequences is the global optimizer and SQP is applied to the best individual to find the local minimum. Noman and Iba [16] proposed an adaptive hill-climbing crossover-based local search operation for enhancing the performance of standard differential evolution (DEahcSPX). Muelas et al. [17] developed MDE-DC which combines DE with multiple trajectory search algorithm (MTS). Neri and Tirronen [18] proposed the scale factor local search differential evolution (SFLSDE). In SFLSDE, golden section search and unidimensional hill-climb local search are applied to detect an optimal value of the scale factor and generate a higher quality offspring. Wang et al. [19] proposed an adaptive MA framework called DE-LS. In DE-LS, self-adaptive differential evolution (SaDE) [20] is the global search method, while covariance matrix adaptation evolution strategy (CMA-ES) [21] and self-adaptive mixed distribution based univariate EDA (MUEDA) [22] are employed as the local search methods. Vasile et al. [10] proposed an inflationary differential evolution algorithm (IDEA), which hybridizes DE with the restarting procedure of Monotonic Basin Hopping (MBH), to solve space trajectory optimization problems. Minisci and Vasile [9] and Di Carlo et al. [8] proposed an adaptive version of inflationary differential evolution algorithm (AIDEA) and a multipopulation version of AIDEA (MP-AIDEA) which automatically adapt the values of four control parameters. Locatelli et al. [23] proposed a memetic differential evolution for disk-packing and sphere-packing problems. In this algorithm, two kinds of local searches (MINOS and SNOPT) are used to detect local minima. Asafuddoula et al. [24] proposed an adaptive hybrid DE algorithm (AH-DEa) which has three features. The first is its use of adaptive crossover rates from a given set of discrete values. The second is an adaptive crossover strategy at different stages of the evolution. The last is the inclusion of a local search strategy to further improve the best solution. Qin et al. [25] proposed an advanced SaDE, which incorporates two different local search chains (Lamarckian and Baldwinian) into SaDE to enhance exploitation capability. Trivedi et al. [26] hybridized DE and GA to solve the unit commitment scheduling problems, in which GA was used to handle the binary unit commitment variables while DE was employed to optimize the continuous power dispatch related variables. In the same year, Li et al. [27] proposed a new hybridization, named DEEP, based on DE framework and the key features of CMA-ES, which generates a trial vector by first using a DE/rand/1/bin strategy followed by an Evolution Path (EP) mutation of CMA-ES.

The focus of this paper is to optimally combine DE global search operators with Broyden-Fletcher-Goldfarb-Shanno (BFGS) algorithm to improve local search in continuous optimization. A new contraction criterion, which is based on the maximum distances in objective space and decision space, is proposed. When the contraction criterion is satisfied, BFGS starts from the best solution at the current generation. Furthermore, a restart mechanism is employed. If the best solution is not improved during the course of the local search, the population is reinitialized to increase the chance to find the global optimum.

The paper is organized as follows: DE is briefly introduced in Section 2. The proposed DE algorithm with local search and reinitialization is presented in Section 3. The design of the experiments, the results, and the corresponding discussions are included in Section 4. The last section, Section 5, is devoted to conclusions and the future work.

## 2. A Short Introduction to Differential Evolution

DE is a population-based stochastic parallel optimization method. Each vector (or individual) of the population at *t* generation is called the target vector, and it will generate one offspring called the trial vector. For example, the *i*th vector of the population *x*_*i*_ will generate one trial vector *u*_*i*_. Trial vectors are generated by adding weighted difference vectors to the target vector. This process is referred to as the mutation operator where the target vector is mutated. A crossover step is then applied to produce an offspring which is only accepted if it improves on the fitness of the parent individual. Many variants of standard DE have been proposed, which use different learning strategies and/or recombination operations in the reproduction stage. A general DE variant may be recorded as DE/a/b/c, where “a” denotes the mutation strategy, “b” specifies the number of difference vectors used, and “c” specifies the crossover scheme which may be binomial or exponential. The DE/rand/1/exp is described in Algorithm 1.

## 3. Proposed Algorithm

In this section, we describe four major operations of the proposed MDE algorithm in detail, including contraction criterion, BFGS search, reinitialization scheme, and boundary constraint handling. The detailed description of MDE is given in Algorithm 2.

### 3.1. Contraction Criterion

In order to design an effective and efficient hybrid algorithm for global optimization, we need to take advantage of both the exploration capabilities of EA and the exploitation capabilities of LS and combine them in a well-balanced manner. To incorporate BFGS into DE successfully, a triggering condition, called contraction criterion, is needed to decide when the local search has to start. There are several kinds of methods to define a contraction criterion. Qin and Suganthan [20] applies local search method after a fixed number of generations (every 200 generations). Sun et al. [5] starts the LS if the promising solution is not updated in t-consecutive generations. Simon et al. [4] use the minimum fitness in the objective space as the contraction criterion; Di Carlo et al. [8–10] perform LS when the maximum distance in decision space is below a given threshold.

In MDE, we propose a new contraction criterion which combines two criteria: (a) *ρ*_1_ is the improved maximum distance in objective space and (b) *ρ*_2_ is the maximum distance in decision space. The idea of *ρ*_1_ is derived from [28]

(1) $$\begin{array}{c} \rho_{1}={\left[\;\sum_{i=1}^{M}\frac{{\left(f_{i}\left(x\right)-f_{avg}\left(x\right)\right)}^{2}}{\left(M-1\right)}\;\right]}^{1/2}, \end{array}$$

where *ρ*_1_ is a measure of the diversity of the population in objective space.

The distance in decision space is defined as

(2) $$\begin{array}{c} \rho_{2}=\max\left(\;\left\|\;x_{i}-x_{j}\;\right\|\;\right),\;\forall x_{i},x_{j}\in P, \end{array}$$

where ||·|| is the Euclidean distance. *ρ*_2_ is a measure of the diversity of the population in decision space.

### 3.2. BFGS Search

In MDE, the local search utilizes the better solutions obtained by the global search to update the population of MDE and thus enhances MDE's exploitation ability to find the best solution. In MDE, we use the BFGS algorithm as the local search method. BFGS is one of the quasi-Newton methods which do not need the precise Hessian matrix and is able to approximate it based on the individual successive gradients. BFGS is considered as the most effective and popular quasi-Newton method and has been proven to have good performance even for nonsmooth optimizations. The details can be found in [29].

### 3.3. Reinitialization Scheme

If the best solution has not been improved after local search, a reinitialization of the whole population is used to give the algorithms more opportunities to find the global optimum. Simon et al. [4] proposed a partial reinitialization of the population. Every 20 generations, the algorithm selects the best *M* individuals from a temporary population of 2*M* + 2 individuals as the reinitialization pool. Sun et al. [5] chose the individuals, which have the largest distances from the local optima, from a temporary population to form the next population. Zamuda et al. [30] proposed a population size reduction method as the reinitialization scheme. In MDE, we apply a simple reinitialization scheme described in Algorithm 3. If the result of the local search does not improve the best individual in the population, a reinitialization of the population is triggered. A counter *C* keeps track of the number of restarts. For *C* < *C*_max_, where *C*_max_ is user-defined, *M* individuals are generated randomly in the search space, drawing samples from a uniform distribution. For *C* ≥ *C*_max_, 2*M*/3 individuals in the population are initialized randomly in the search space, while the rest are initialized by a normal distribution which takes the best individual as the center and (*U*_*i*_ − *L*_*i*_)/50 as the standard deviation. Algorithm 3 summarises the reinitialization procedure. The function* randreal* draws samples from a uniform distribution while function* Gaussian* draws samples from a normal distribution and [*L*_*i*_, *U*_*i*_] are the lower and upper boundaries on *x*_*i*_.

### 3.4. Boundary Constraint Handling

After mutation and crossover, each generated trial vector *u*_*i*_ undergoes boundary constraint check. If some variables of *u*_*i*_ are out of the boundary, a repair method is applied as follows:

(3) $$\begin{array}{c} x_{i}=randreal\left(\;L_{i},U_{i}\;\right),\;if\;\;x_{i}<L_{i}\;\;or\;\;x_{i}>U_{i}, \end{array}$$

where randreal (*L*_*i*_, *U*_*i*_) can generate a random real number from [*L*_*i*_, *U*_*i*_].

## 4. Experimental Results

In order to verify the performance of MDE, we select the 21 nonnoisy benchmark functions from CEC2005 special session on real-parameter optimization (excluding noisy functions *F*4, *F*17, *F*24, and *F*25) since MDE has no ability to handle functions with noisy landscapes. The details about these functions can be found in [31]. We compare MDE with six peer algorithms, including CLPSO [32], GL-25 [33], CMA-ES [21], LBBO [4], SFLSDE [18], and L-SHADE [34].

### 4.1. Experimental Setup

For each algorithm on each benchmark problem, we conduct 25 independent runs and limit each run to 10000*∗D* max function evaluations, where *D* is the problem dimension (*D* = 10, *D* = 30, and *D* = 50). The performance of the algorithms is evaluated in terms of* function error value* [31], defined as *f*(*x*) − *f*(*x*^*∗*^), where *x*^*∗*^ is the global optimum of the test function. The mean and the standard deviation of the* function error values* are recorded. The parameters of MDE are set as *M* = 30, *ρ*_1,max_ = 2.0, *ρ*_2,max_ = 2.0, *C*_max_ = 3, *CR* ∈ *N*(0.8,0.1), and *F* ∈ *N*(0.5,0.1); the mutation and crossover strategies are the same as those in [24]. For the other six algorithms, we use the same parameter settings in their original papers.

### 4.2. Performance Criteria

To effectively analyze the results, two nonparametric statistical tests, as similarly done in [35, 36], are used in the experiments. (i) Wilcoxon's signed-rank test at *α* = 0.05 is performed to test the statistical significance of the experimental results between two algorithms on both single-problem and multiproblem. (ii) Friedman's test is employed to obtain the average rankings of all the compared algorithms. Wilcoxon's signed-rank test on single-problem is calculated by Matlab, while Wilcoxon's signed-rank test on multiproblem and Friedman test are calculated by the software of KEEL [37].

### 4.3. Comparison between the Other Six Algorithms and MDE

**Figure 1:**
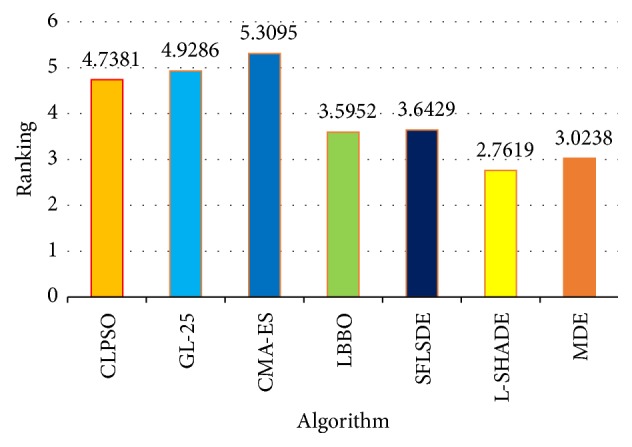
Average rankings of the seven algorithms by Friedman test for all functions at *D* = 10.

Table 1 shows the results of MDE and the other six algorithms on the 10-dimensional benchmarks. It can be seen that MDE performs significantly better than CLPSO, GL-25, CMA-ES, LBBO, SFLSDE, and L-SHADE on 15, 16, 17, 7, 8, and 8 test functions. And CLPSO, GL-25, CMA-ES, LBBO, SFLSDE, and L-SHADE win on 4, 4, 3, 5, 8, and 8 test functions, respectively. MDE obtains similar results with the other six algorithms in 2, 1, 1, 9, 5, and 5 cases. Additionally, the results of the multiple-problem statistical analysis are shown in Table 4. It can be seen that MDE can obtain higher *R*^+^ values than *R*^−^ values in all cases, where *R*^+^ is the sum of ranks for the functions on which MDE outperformed the compared algorithm, and *R*^−^ is the sum of ranks for the opposite [36]. According to Wilcoxon's test at *α* = 0.05 and *α* = 0.1, there are significant differences in three cases (MDE versus CLPSO, MDE versus GL-25, and MDE versus CMA-ES), which means that in those cases MDE is significantly better than CLPSO, GL-25, and CMA-ES. In addition, Friedman's test is employed to evaluate the significant differences of all the compared algorithms. As shown in Figure 1, MDE gets the second average ranking value, while L-SHADE gets the first average ranking values on the 10-dimensional problems.

**Figure 2:**
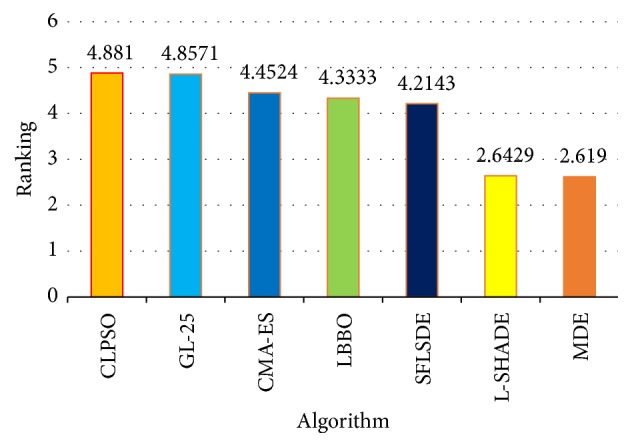
Average rankings of the seven algorithms by Friedman test for all functions at *D* = 30.

Table 2 shows that MDE performs significantly better than the other six compared algorithms in the majority of the test functions. For example, MDE wins in 12 cases, only loses in 3 cases, and ties in 6 cases, compared with SFLSDE. We can also find that MDE obtains much better solutions than LBBO and CLPSO. As for L-SHADE, MDE wins in 8 cases and loses in 10 cases. According to the results of the multiple-problem statistical analysis shown in Table 5, it can be seen that MDE can obtain higher *R*^+^ values than *R*^−^ values in all cases. According to Wilcoxon's test at *α* = 0.05 and *α* = 0.1, there are significant differences in four cases (MDE versus CLPSO, MDE versus GL-25, MDE versus CMA-ES, and MDE versus SFLSDE), which means that in those cases MDE is significantly better than CLPSO, GL-25, CMA-ES, and SFLSDE. And from Table 5, we can find that L-SHADE and MDE have comparable results. Moreover, Figure 2 shows that MDE performs the first average ranking value and L-SHADE obtains the second average ranking values on the 30-dimensional problems by Friedman's test.

**Figure 3:**
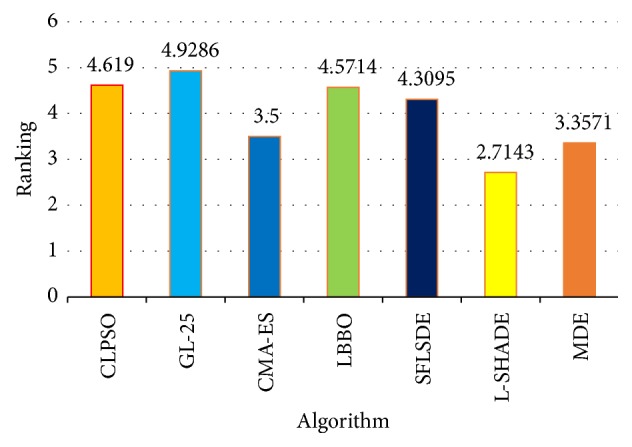
Average rankings of the seven algorithms by Friedman test for all functions at *D* = 50.

Table 3 shows that MDE also performs significantly better than five compared algorithms in the majority of the test functions except L-SHADE. For example, MDE wins in 15 cases, only loses in 3 cases, and ties in 3 cases, compared with LBBO. When comparing with L-SHADE, MDE wins in 8 cases and loses in 12 cases.  Table 6 shows that MDE can perform higher *R*^+^ values than *R*^−^ values in five cases. According to Wilcoxon's test at *α* = 0.05 and *α* = 0.1, there are significant differences in two cases (MDE versus GL-25 and MDE versus LBBO), which means that in those cases MDE is significantly better than GL-25 and LBBO.  Figure 3 shows that L-SHADE obtains better average ranking values than the other six algorithms on 50-dimensional problems by Friedman's test.

In general, according to the analysis above, we can conclude that MDE and L-SHADE have better average rankings among the seven algorithms on 21 benchmark problems for all of three different dimensions. The performance of MDE is comparable to L-SHADE on 10- and 30-dimensional problems, while L-SHADE is better than MDE on 50-dimensional problems because of the larger initial population and linear population size reduction.

### 4.4. Influence of Contraction Criterion

In the previous experiments the recommended initial *ρ*_1,max_ = 2.0 and *ρ*_2,max_ = 2.0 are used. In order to test the influence of different initial contraction criterion values to the enhanced performance of MDE, in this section, MDE is tested with different initial *ρ*_1,max_ and *ρ*_2,max_ values. The initial values are set as *ρ*_1,max_ = {1.0,2.0,3.0} and *ρ*_2,max_ = {1.0,2.0,3.0}. All other parameters are not changed as described in Section 4.1. Nine groups of experiments with different combinations between *ρ*_1,max_ and *ρ*_2,max_ are done. *ρ*_1.0,1.0_ means the value of parameters *ρ*_1,max_ = 1.0 and *ρ*_2,max_ = 1.0. The statistical results by Friedman's test with all initial values are shown in Table 7.

From Table 7, MDE owes the best average ranking value at *ρ*_2.0,2.0_ than the other 8 groups on both 10-dimensional and 30-dimensional test functions. On 50-dimensional test functions, *ρ*_1.0,1.0_ is the better choice to MDE. In general, we can conclude that it is better to set smaller contraction criterion values as the dimension of test functions increases.

### 4.5. Influence of Parameter *C*_max_

The experiment is to test the influence of parameter *C*_max_ in MDE. Friedman's test results are shown in Table 8, where the values of *C*_max_ are set as  *C*_max_ = {3,5, 7,9, 11,13,15} in Table 8. All other parameters are kept unchanged as described in Section 4.1. In addition, all experiments are conducted for 25 independent runs for each function.

It can be seen from Table 8 that MDE with *C*_max_ = 3.0 gets the better average ranking value than the other six cases at *D* = 10. At *D* = 30, *C*_max_ = 5.0 is the best choice and *C*_max_ = 3.0 is the second best choice to MDE. On 50-dimensional test functions, *C*_max_ = 3.0 is the better choice parameter to MDE. Generally speaking, the small *C*_max_ value such as 3 or 5 is good to enhance the performance of the MDE algorithm.

### 4.6. Influence of Population Size *M*

To analyze the influence of the population size *M*, different values of *M* are tested in a set of experiments. Friedman's test results are shown in Table 9, where the values of *M* are set as {30,60,90,120,150}. All other parameters are kept unchanged as described in Section 4.1.

From Table 9, MDE with *M* = 30 ranks the first both at *D* = 10 and *D* = 30, while MDE with *M* = 90 performs the best at *D* = 50. From the results, it can be concluded that, as the dimension increases, a properly increased population size *M* can enhance the search capability of MDE.

## 5. Conclusion

A memetic differential evolution, MDE, has been introduced in this paper. MDE uses a new contraction criterion to decide when the local search starts. In addition, MDE includes the global and local search operators, along with reinitialization, to improve performance.

To evaluate the performance of MDE, 21 benchmark functions with different characteristics are chosen for test. The results show that (i) MDE can obtain better or at least comparable results, compared with the other six algorithms; (ii) small contraction criterion values and *C*_max_ can enhance the performance of MDE in terms of the quality of the final results; (iii) a large population size is good to MDE as the dimension increases.

In this paper, some preliminary experiments have been performed to verify its effect on the performance of MDE. In our future work, MDE will be tested on some real-world applications problems. Moreover, we believe that some other local search algorithms and adaptive population size strategy can also be used in MDE.

## Figures and Tables

**Algorithm 1:**
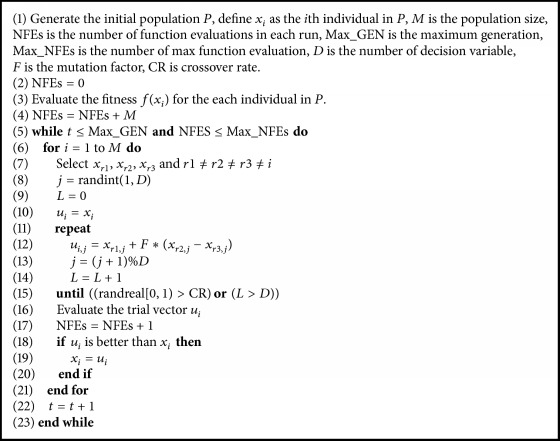
DE with rand/1/exp.

**Algorithm 2:**
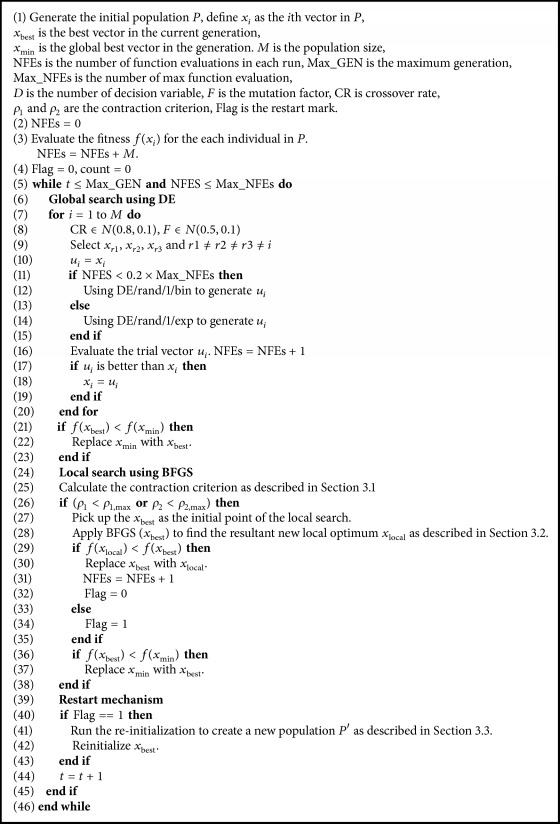
Pseudocode of MDE.

**Algorithm 3:**
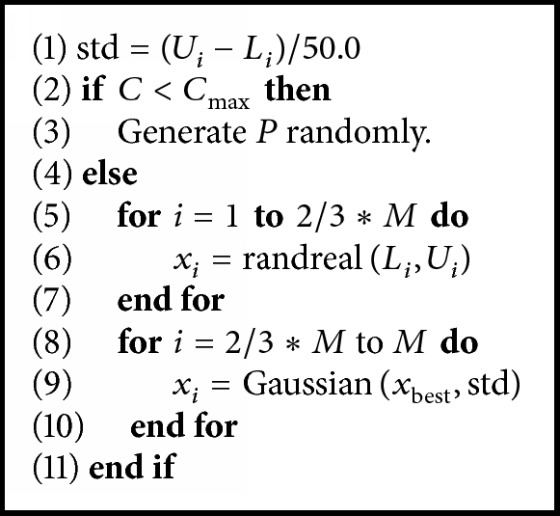
Pseudocode of reinitialization scheme.

**Table 1:** Comparison of Mean Error and standard deviation between MDE and other six EAs over 25 independent runs on twenty-one 10-dimensional test functions.

Prob	CLPSO	GL-25	CMA-ES	LBBO	SFLSDE	L-SHADE	MDE
F01^*∗*^	3.32*E* + 00 ± 1.96*E* + 00−	1.71*E* − 26 ± 4.40*E* − 26−	3.17*E* − 27 ± 1.82*E* − 27−	0.00*E* + 00 ± 0.00*E* + 00=	6.50*E* − 09 ± 1.37*E* − 08−	6.66*E* − 08 ± 1.05*E* − 07−	0.00*E* + 00 ± 0.00*E* + 00
F02	7.42*E* − 02 ± 5.95*E* − 02−	2.73*E* − 28 ± 4.79*E* − 28−	6.17*E* − 27 ± 2.18*E* − 27−	0.00*E* + 00 ± 0.00*E* + 00=	0.00*E* + 00 ± 0.00*E* + 00=	0.00*E* + 00 ± 0.00*E* + 00=	0.00*E* + 00 ± 0.00*E* + 00
F03	3.54*E* + 05 ± 1.45*E* + 05−	2.49*E* + 04 ± 1.12*E* + 04−	3.94*E* − 23 ± 2.17*E* − 23−	0.00*E* + 00 ± 0.00*E* + 00=	9.54*E* + 03 ± 8.05*E* + 03−	0.00*E* + 00 ± 0.00*E* + 00=	0.00*E* + 00 ± 0.00*E* + 00
F05	6.29*E* + 00 ± 5.49*E* + 00−	5.21*E* − 05 ± 2.28*E* − 04+	4.82*E* − 11 ± 6.54*E* − 12+	3.06*E* − 03 ± 3.68*E* − 03+	0.00*E* + 00 ± 0.00*E* + 00+	0.00*E* + 00 ± 0.00*E* + 00+	1.13*E* − 02 ± 8.32*E* − 03
F06	9.25*E* − 01 ± 7.47*E* − 01−	2.30*E* + 00 ± 6.43*E* − 01−	9.57*E* − 01 ± 1.74*E* + 00−	1.53*E* − 06 ± 4.89*E* − 06=	1.59*E* − 01 ± 7.97*E* − 01=	0.00*E* + 00 ± 0.00*E* + 00=	0.00*E* + 00 ± 0.00*E* + 00
F07	2.82*E* − 01 ± 9.68*E* − 02−	1.07*E* − 01 ± 2.80*E* − 02−	1.21*E* − 02 ± 1.16*E* − 02+	1.06*E* − 01 ± 2.15*E* − 01=	1.27*E* + 03 ± 1.70*E* − 01−	3.84*E* − 03 ± 6.20*E* − 03+	1.87*E* − 02 ± 1.75*E* − 02
F08	2.04*E* + 01 ± 8.71*E* − 02−	2.04*E* + 01 ± 8.87*E* − 02−	2.00*E* + 01 ± 7.49*E* − 02=	2.00*E* + 01 ± 2.95*E* − 09=	2.04*E* + 01 ± 9.14*E* − 02−	2.01*E* + 01 ± 1.03*E* − 01−	2.00*E* + 01 ± 0.00*E* + 00
F09^*∗*^	5.47*E* + 00 ± 1.61*E* + 00−	6.83*E* + 00 ± 4.58*E* + 00−	1.26*E* + 02 ± 4.21*E* + 01−	9.80*E* − 12 ± 1.31*E* − 11+	2.91*E* + 00 ± 2.27*E* + 00+	8.37*E* − 01 ± 7.90*E* − 01+	4.54*E* + 00 ± 1.99*E* + 00
F10	8.53*E* + 00 ± 1.70*E* + 00−	1.36*E* + 01 ± 9.53*E* + 00−	7.51*E* + 01 ± 1.01*E* + 02−	1.32*E* + 01 ± 3.76*E* + 00−	7.41*E* + 00 ± 2.95*E* + 00−	2.47*E* + 00 ± 1.04*E* + 00+	4.10*E* + 00 ± 1.16*E* + 00
F11	5.01*E* + 00 ± 7.30*E* − 01+	3.20*E* + 00 ± 9.45*E* − 01+	2.42*E* + 00 ± 1.30*E* + 00+	5.04*E* + 00 ± 1.17*E* + 00+	1.77*E* + 00 ± 1.36*E* + 00+	4.12*E* + 00 ± 7.50*E* − 01+	6.69*E* + 00 ± 1.56*E* + 00
F12	1.38*E* + 02 ± 9.66*E* + 01−	1.04*E* + 01 ± 8.81*E* − 01−	6.92*E* + 03 ± 1.21*E* + 04−	2.59*E* − 02 ± 1.20*E* − 01−	1.15*E* + 02 ± 3.33*E* + 02−	2.40*E* + 00 ± 4.36*E* + 00−	0.00*E* + 00 ± 0.00*E* + 00
F13	4.56*E* − 01 ± 7.95*E* − 02−	9.68*E* − 01 ± 4.21*E* − 01−	1.02*E* + 00 ± 4.47*E* − 01−	4.16*E* − 01 ± 1.52*E* − 01=	3.66*E* − 01 ± 1.02*E* − 01+	2.31*E* − 01 ± 3.67*E* − 02+	4.05*E* − 01 ± 1.26*E* − 01
F14	3.15*E* + 00 ± 2.24*E* − 01+	2.86*E* + 00 ± 4.76*E* − 01+	4.87*E* + 00 ± 1.87*E* − 01−	3.33*E* + 00 ± 3.55*E* − 01+	2.86*E* + 00 ± 3.29*E* − 01+	2.40*E* + 00 ± 2.93*E* − 01+	3.77*E* + 00 ± 3.30*E* − 01
F15	9.30*E* + 00 ± 2.09*E* + 01+	3.79*E* + 02 ± 6.21*E* + 01−	5.30*E* + 02 ± 2.76*E* + 02−	1.70*E* − 12 ± 1.89*E* − 12+	2.72*E* + 02 ± 1.53*E* + 02−	1.78*E* + 02 ± 1.90*E* + 02−	6.04*E* + 01 ± 7.57*E* + 01
F16	1.15*E* + 02 ± 1.04*E* + 01=	9.69*E* + 01 ± 1.10*E* + 01+	2.03*E* + 02 ± 2.23*E* + 02−	1.27*E* + 02 ± 1.51*E* + 01−	1.11*E* + 02 ± 1.10*E* + 01+	9.26*E* + 01 ± 2.66*E* + 00+	1.15*E* + 02 ± 1.17*E* + 01
F18	6.57*E* + 02 ± 1.18*E* + 02−	7.88*E* + 02 ± 5.80*E* + 01−	8.26*E* + 02 ± 3.85*E* + 02−	7.60*E* + 02 ± 1.61*E* + 02−	5.20*E* + 02 ± 2.55*E* + 02+	6.00*E* + 02 ± 2.50*E* + 02=	5.51*E* + 02 ± 2.36*E* + 02
F19	6.11*E* + 02 ± 1.39*E* + 02−	8.00*E* + 02 ± 1.86*E* − 02−	7.73*E* + 02 ± 3.44*E* + 02−	7.71*E* + 02 ± 1.49*E* + 02−	5.33*E* + 02 ± 2.21*E* + 02=	6.40*E* + 02 ± 2.38*E* + 02−	4.95*E* + 02 ± 2.38*E* + 02
F20	6.64*E* + 02 ± 1.52*E* + 02−	7.80*E* + 02 ± 1.00*E* + 02−	7.06*E* + 02 ± 2.86*E* + 02−	7.11*E* + 02 ± 1.97*E* + 02−	4.54*E* + 02 ± 2.15*E* + 02+	6.20*E* + 02 ± 2.45*E* + 02−	5.17*E* + 02 ± 2.37*E* + 02
F21	4.49*E* + 02 ± 1.11*E* + 02=	8.00*E* + 02 ± 8.37*E* − 14−	8.54*E* + 02 ± 2.64*E* + 02−	5.35*E* + 02 ± 2.66*E* + 02−	5.64*E* + 02 ± 1.89*E* + 02−	4.88*E* + 02 ± 1.90*E* + 02−	4.45*E* + 02 ± 1.83*E* + 02
F22	7.47*E* + 02 ± 9.64*E* + 01−	6.72*E* + 02 ± 1.90*E* + 02=	7.69*E* + 02 ± 2.53*E* + 01−	6.51*E* + 02 ± 2.24*E* + 02=	7.63*E* + 02 ± 2.85*E* + 01=	7.45*E* + 02 ± 1.20*E* + 01=	6.90*E* + 02 ± 1.64*E* + 02
F23	5.58*E* + 02 ± 6.83*E* + 01+	9.74*E* + 02 ± 1.63*E* + 01−	9.89*E* + 02 ± 2.59*E* + 02−	6.40*E* + 02 ± 9.69*E* + 01=	6.62*E* + 02 ± 1.87*E* + 02=	6.44*E* + 02 ± 1.24*E* + 02−	6.42*E* + 02 ± 1.46*E* + 02

−	15	16	17	7	8	8	/
+	4	4	3	5	8	8	/
=	2	1	1	9	5	5	/

*∗* indicates that when six algorithms obtain the global optimum, the intermediate results are reported at NFEs = 10000. “−,” “+,” and “=” denote that the performance of this algorithm is, respectively, worse than, better than, and similar to MDE according to the Wilcoxon signed-rank test at *α* = 0.05.

**Table 2:** Comparison of Mean Error and standard deviation between MDE and other six EAs over 25 independent runs on twenty-one 30-dimensional test functions.

Prob	CLPSO	GL-25	CMA-ES	LBBO	SFLSDE	L-SHADE	MDE
F01^*∗*^	2.81*E* + 01 ± 6.52*E* + 00−	7.05*E* − 24 ± 3.18*E* − 23=	2.05*E* − 25 ± 6.01*E* − 26=	0.00*E* + 00 ± 0.00*E* + 00+	1.36*E* − 11 ± 1.43*E* − 11−	3.52*E* − 05 ± 2.87*E* − 05−	1.36*E* − 14 ± 2.48*E* − 14
F02	8.66*E* + 02 ± 1.75*E* + 02−	5.46*E* + 01 ± 8.48*E* + 01−	6.36*E* − 25 ± 2.18*E* − 25+	2.11*E* − 08 ± 8.29*E* − 08−	3.78*E* − 09 ± 4.84*E* − 09−	6.82*E* − 15 ± 1.89*E* − 14+	2.07*E* − 13 ± 8.51*E* − 14
F03	1.62*E* + 07 ± 4.99*E* + 06−	2.13*E* + 06 ± 8.45*E* + 05−	5.38*E* − 21 ± 1.66*E* − 21−	3.97*E* + 02 ± 6.54*E* + 02−	3.97*E* + 05 ± 2.40*E* + 05−	2.86*E* − 13 ± 5.04*E* − 13−	0.00*E* + 00 ± 0.00*E* + 00
F05	3.97*E* + 03 ± 4.11*E* + 02−	2.48*E* + 03 ± 2.08*E* + 02−	3.34*E* − 10 ± 7.85*E* − 11+	2.69*E* + 03 ± 7.88*E* + 02−	1.07*E* + 03 ± 6.35*E* + 02−	1.32*E* − 11 ± 7.71*E* − 12+	3.05*E* + 02 ± 9.81*E* + 01
F06	6.09*E* + 00 ± 5.44*E* + 00−	2.17*E* + 01 ± 1.53*E* + 00−	4.78*E* − 01 ± 1.32*E* + 00=	2.15*E* − 01 ± 1.03*E* + 00−	4.78*E* − 01 ± 1.32*E* + 00−	1.00*E* − 13 ± 5.51*E* − 14−	0.00*E* + 00 ± 0.00*E* + 00
F07	4.85*E* − 01 ± 8.90*E* − 02−	1.37*E* − 02 ± 1.10*E* − 02−	1.58*E* − 03 ± 4.92*E* − 03−	9.77*E* − 02 ± 2.66*E* − 01−	4.70*E* + 03 ± 1.73*E* + 00−	0.00*E* + 00 ± 0.00*E* + 00+	6.71*E* − 14 ± 2.83*E* − 14
F08	2.10*E* + 01 ± 5.95*E* − 02−	2.10*E* + 01 ± 5.19*E* − 02−	2.03*E* + 01 ± 5.70*E* − 01−	2.00*E* + 01 ± 1.25*E* − 04−	2.10*E* + 01 ± 4.53*E* − 02−	2.03*E* + 01 ± 3.47*E* − 01−	2.00*E* + 01 ± 0.00*E* + 00
F09^*∗*^	3.21*E* + 01 ± 5.26*E* + 00−	4.84*E* + 01 ± 3.62*E* + 01−	4.28*E* + 02 ± 1.13*E* + 02−	2.48*E* − 07 ± 3.31*E* − 07+	3.37*E* + 01 ± 7.65*E* + 00−	4.10*E* + 01 ± 5.83*E* + 00−	2.69*E* + 01 ± 1.11*E* + 01
F10	1.05*E* + 02 ± 1.35*E* + 01−	1.74*E* + 02 ± 1.22*E* + 01−	4.95*E* + 01 ± 1.29*E* + 01−	1.73*E* + 02 ± 3.10*E* + 01−	4.34*E* + 01 ± 1.27*E* + 01=	6.53*E* + 00±1.60*E* + 00+	4.07*E* + 01 ± 7.91*E* + 00
F11	2.56*E* + 01 ± 1.55*E* + 00+	3.31*E* + 01 ± 7.73*E* + 00−	7.43*E* + 00 ± 2.36*E* + 00+	2.63*E* + 01 ± 2.75*E* + 00+	1.72*E* + 01 ± 3.30*E* + 00+	2.64*E* + 01 ± 1.33*E* + 00+	2.80*E* + 01 ± 3.08*E* + 00
F12	1.78*E* + 04 ± 5.59*E* + 03−	7.13*E* + 03 ± 5.05*E* + 03−	1.11*E* + 04 ± 9.70*E* + 03−	1.55*E* + 01 ± 3.49*E* + 01=	9.72*E* + 03 ± 8.88*E* + 03−	8.97*E* + 02 ± 1.27*E* + 03−	1.93*E* + 02 ± 4.79*E* + 02
F13	2.12*E* + 00 ± 2.68*E* − 01−	5.28*E* + 00 ± 4.08*E* + 00−	3.54*E* + 00 ± 7.71*E* − 01−	1.94*E* + 00 ± 4.23*E* − 01−	2.02*E* + 00 ± 5.91*E* − 01−	1.24*E* + 00 ± 1.06*E* − 01+	1.55*E* + 00 ± 3.51*E* − 01
F14	1.28*E* + 01 ± 1.80*E* − 01+	1.29*E* + 01 ± 4.63*E* − 01+	1.46*E* + 01 ± 2.91*E* − 01−	1.29*E* + 01 ± 2.63*E* − 01+	1.27*E* + 01 ± 5.54*E* − 01+	1.18*E* + 01 ± 3.43*E* − 01+	1.38*E* + 01 ± 3.18*E* − 01
F15	6.15*E* + 01 ± 5.70*E* + 01+	3.04*E* + 02 ± 2.00*E* + 01=	4.09*E* + 02 ± 2.21*E* + 02=	7.90*E* + 01 ± 1.37*E* + 02+	3.04*E* + 02 ± 9.27*E* + 01=	3.84*E* + 02 ± 4.73*E* + 01−	3.23*E* + 02 ± 1.02*E* + 02
F16	1.71*E* + 02 ± 2.88*E* + 01−	1.28*E* + 02 ± 9.13*E* + 01=	4.32*E* + 02 ± 3.57*E* + 02−	1.74*E* + 02 ± 3.52*E* + 01−	1.48*E* + 02 ± 1.19*E* + 02=	2.39*E* + 01 ± 2.60*E* + 00+	9.89*E* + 01±2.61*E* + 01
F18	8.97*E* + 02 ± 7.87*E* + 01−	9.07*E* + 02 ± 1.37*E* + 00=	9.28*E* + 02 ± 1.18*E* + 02=	9.16*E* + 02 ± 3.51*E* + 01−	9.05*E* + 02 ± 1.36*E* + 00=	9.03*E* + 02 ± 1.81*E* − 01=	8.91*E* + 02 ± 4.10*E* + 01
F19	9.14*E* + 02 ± 1.73*E* + 00−	9.06*E* + 02 ± 1.48*E* + 00=	9.04*E* + 02 ± 2.83*E* − 01=	9.21*E* + 02 ± 2.53*E* + 01−	9.06*E* + 02 ± 1.83*E* + 00=	9.03*E* + 02 ± 1.94*E* − 01=	8.93*E* + 02 ± 4.18*E* + 01
F20	9.14*E* + 02 ± 1.23*E* + 00−	9.07*E* + 02 ± 1.49*E* + 00=	9.21*E* + 02 ± 8.59*E* + 01=	9.24*E* + 02 ± 3.14*E* + 00−	9.06*E* + 02 ± 4.11*E* + 00=	9.03*E* + 02 ± 2.07*E* − 01=	9.02*E* + 02 ± 3.18*E* + 01
F21	5.00*E* + 02 ± 5.42*E* − 13−	5.00*E* + 02 ± 4.27*E* − 13−	5.12*E* + 02 ± 6.00*E* + 01−	5.00*E* + 02 ± 1.07*E* − 08−	5.04*E* + 02 ± 1.75*E* + 01−	5.00*E* + 02 ± 2.89*E* − 13−	5.00*E* + 02 ± 2.54*E* − 13
F22	9.70*E* + 02 ± 1.17*E* + 01−	9.27*E* + 02 ± 8.14*E* + 00+	8.27*E* + 02 ± 1.82*E* + 01+	1.06*E* + 03 ± 3.65*E* + 01−	8.71*E* + 02 ± 1.88*E* + 01+	8.42*E* + 02 ± 1.81*E* + 01+	9.33*E* + 02 ± 1.40*E* + 01
F23	5.34*E* + 02 ± 1.15*E* − 04+	5.34*E* + 02 ± 4.63*E* − 04+	5.37*E* + 02 ± 4.41*E* + 00−	5.90*E* + 02 ± 9.52*E* + 00−	7.07*E* + 02 ± 1.57*E* + 02−	5.34*E* + 02±5.77*E* − 13+	5.34*E* + 02 ± 5.38*E* − 04

−	17	12	11	15	12	8	/
+	4	3	4	5	3	10	/
=	0	6	6	1	6	3	/

*∗* indicates that when six algorithms obtain the global optimum, the intermediate results are reported at NFEs = 30000. “−,” “+,” and “=” denote that the performance of this algorithm is, respectively, worse than, better than, and similar to MDE according to the Wilcoxon signed-rank test at *α* = 0.05.

**Table 3:** Comparison of Mean Error and standard deviation between MDE and other six EAs over 25 independent runs on twenty-one 50-dimensional test functions.

Prob	CLPSO	GL-25	CMA-ES	LBBO	SFLSDE	L-SHADE	MDE
F01	0.00*E* + 00 ± 0.00*E* + 00=	1.48*E* − 23 ± 5.81*E* − 23−	4.46*E* − 25 ± 8.18*E* − 26−	1.46*E* − 10 ± 1.23*E* − 10−	4.77*E* − 14 ± 2.13*E* − 14−	0.00*E* + 00 ± 0.00*E* + 00=	0.00*E* + 00 ± 0.00*E* + 00
F02	8.94*E* + 03 ± 1.24*E* + 03−	1.54*E* + 03 ± 1.10*E* + 03−	6.51*E* − 24 ± 1.70*E* − 24+	4.42*E* − 07 ± 2.80*E* − 07−	1.24*E* + 00 ± 8.43*E* − 01−	2.11*E* − 13 ± 5.81*E* − 14+	2.73*E* − 13 ± 9.14*E* − 14
F03	4.35*E* + 07 ± 1.05*E* + 07−	5.50*E* + 06 ± 2.00*E* + 06−	4.26*E* − 20 ± 8.09*E* − 21−	1.57*E* + 04 ± 8.05*E* + 03−	1.48*E* + 06 ± 5.79*E* + 05−	1.26*E* + 03 ± 1.44*E* + 03−	0.00*E* + 00 ± 0.00*E* + 00
F05	9.45*E* + 03 ± 8.94*E* + 02−	5.70*E* + 03 ± 5.04*E* + 02−	1.40*E* − 01 ± 6.98*E* − 01+	8.53*E* + 03 ± 1.58*E* + 03−	3.33*E* + 03 ± 7.16*E* + 02−	2.09*E* + 02 ± 2.02*E* + 02+	1.19*E* + 03 ± 4.08*E* + 02
F06	1.43*E* + 01 ± 1.54*E* + 01−	4.95*E* + 01 ± 2.14*E* + 01−	4.78*E* − 01 ± 1.32*E* + 00=	3.97*E* + 01 ± 7.63*E* + 01−	2.72*E* + 01 ± 3.19*E* + 01−	1.30*E* − 01 ± 3.71*E* − 01−	0.00*E* + 00 ± 0.00*E* + 00
F07	3.58*E* − 01 ± 5.69*E* − 02−	6.40*E* − 02 ± 5.49*E* − 02−	1.58*E* − 03 ± 4.38*E* − 03−	4.78*E* − 01 ± 3.03*E* − 01−	6.20*E* + 03 ± 7.88*E* − 13−	2.84*E* − 14 ± 0.00*E* + 00+	2.17*E* − 13 ± 5.31*E* − 14
F08	2.11*E* + 01 ± 4.65*E* − 02−	2.11*E* + 01 ± 4.06*E* − 02−	2.05*E* + 01 ± 7.13*E* − 01−	2.00*E* + 01 ± 1.16*E* − 02−	2.11*E* + 01 ± 3.53*E* − 02−	2.04*E* + 01 ± 4.43*E* − 01−	2.00*E* + 01 ± 0.00*E* + 00
F09	0.00*E* + 00 ± 0.00*E* + 00=	5.37*E* + 01 ± 1.23*E* + 01−	6.87*E* + 02 ± 1.74*E* + 02−	9.05*E* − 05 ± 2.41*E* − 04=	4.38*E* − 01 ± 1.12*E* + 00−	5.56*E* − 09 ± 9.78*E* − 09−	0.00*E* + 00 ± 0.00*E* + 00
F10	2.62*E* + 02 ± 3.16*E* + 01−	2.41*E* + 02 ± 1.47*E* + 02−	9.56*E* + 01 ± 1.92*E* + 01=	3.70*E* + 02 ± 4.89*E* + 01−	9.15*E* + 01 ± 4.17*E* + 01+	1.28*E* + 01 ± 2.30*E* + 00+	9.78*E* + 01 ± 1.51*E* + 01
F11	5.09*E* + 01 ± 2.13*E* + 00+	6.49*E* + 01 ± 1.08*E* + 01−	1.15*E* + 01 ± 3.69*E* + 00+	5.45*E* + 01 ± 2.84*E* + 00+	4.22*E* + 01 ± 1.62*E* + 01+	5.23*E* + 01 ± 2.12*E* + 00+	5.90*E* + 01 ± 4.90*E* + 00
F12	6.73*E* + 04 ± 1.39*E* + 04−	4.40*E* + 04 ± 2.00*E* + 04−	3.03*E* + 04 ± 2.59*E* + 04−	1.01*E* + 03 ± 1.26*E* + 03=	2.75*E* + 04 ± 2.29*E* + 04−	6.89*E* + 03 ± 6.05*E* + 03−	1.35*E* + 03 ± 2.45*E* + 03
F13	3.75*E* + 00 ± 4.16*E* − 01−	1.13*E* + 01 ± 8.61*E* + 00−	6.00*E* + 00 ± 1.57*E* + 00−	4.03*E* + 00 ± 4.28*E* − 01−	8.90*E* + 00 ± 4.83*E* − 01−	2.61*E* + 00 ± 1.45*E* − 01+	2.96*E* + 00 ± 4.38*E* − 01
F14	2.25*E* + 01 ± 2.06*E* − 01+	2.27*E* + 01 ± 1.81*E* − 01+	2.44*E* + 01 ± 3.08*E* − 01−	2.23*E* + 01 ± 3.79*E* − 01+	2.28*E* + 01 ± 3.26*E* − 01+	2.11*E* + 01 ± 5.41*E* − 01+	2.36*E* + 01 ± 2.62*E* − 01
F15	8.87*E* + 01 ± 6.41*E* + 01+	3.84*E* + 02 ± 5.52*E* + 01−	3.97*E* + 02 ± 2.27*E* + 02=	2.26*E* + 01 ± 7.66*E* + 01+	2.60*E* + 02 ± 8.66*E* + 01+	3.52*E* + 02 ± 8.72*E* + 01−	3.20*E* + 02 ± 5.52*E* + 01
F16	2.30*E* + 02 ± 4.41*E* + 01−	1.66*E* + 02 ± 9.88*E* + 01−	2.87*E* + 02 ± 2.48*E* + 02−	2.15*E* + 02 ± 2.50*E* + 01−	8.52*E* + 01 ± 3.88*E* + 01+	1.85*E* + 01 ± 1.34*E* + 00+	1.35*E* + 02 ± 5.19*E* + 01
F18	9.46*E* + 02 ± 5.26*E* + 00+	9.24*E* + 02 ± 1.58*E* + 00+	9.12*E* + 02 ± 4.86*E* − 01+	9.65*E* + 02 ± 2.06*E* + 01−	9.17*E* + 02 ± 4.10*E* + 00+	9.13*E* + 02 ± 9.02*E* − 01+	9.56*E* + 02 ± 3.41*E* + 01
F19	9.44*E* + 02 ± 6.29*E* + 00+	9.19*E* + 02 ± 2.49*E* + 01+	9.12*E* + 02 ± 4.87*E* − 01+	9.59*E* + 02 ± 1.33*E* + 01−	9.18*E* + 02 ± 3.14*E* + 00+	9.13*E* + 02 ± 1.91*E* + 00+	9.52*E* + 02 ± 3.31*E* + 01
F20	9.44*E* + 02 ± 4.61*E* + 00+	9.24*E* + 02 ± 1.90*E* + 00+	9.12*E* + 02 ± 4.54*E* − 01+	9.63*E* + 02 ± 2.03*E* + 01=	9.17*E* + 02 ± 3.92*E* + 00+	9.14*E* + 02 ± 1.74*E* + 00+	9.58*E* + 02 ± 7.97*E* + 00
F21	5.00*E* + 02 ± 7.54*E* − 13−	5.00*E* + 02 ± 2.11*E* − 11−	5.68*E* + 02 ± 1.98*E* + 02−	5.00*E* + 02 ± 1.99*E* − 08−	1.01*E* + 03 ± 2.11*E* + 00−	1.00*E* + 03 ± 1.41*E* + 00−	5.00*E* + 02 ± 3.28*E* − 13
F22	1.00*E* + 03 ± 7.79*E* + 00−	9.69*E* + 02 ± 6.73*E* + 00+	8.58*E* + 02 ± 1.06*E* + 01+	1.09*E* + 03 ± 2.52*E* + 01−	8.98*E* + 02 ± 1.31*E* + 01+	8.75*E* + 02 ± 3.13*E* + 00+	9.93*E* + 02 ± 1.51*E* + 01
F23	5.39*E* + 02 ± 8.67*E* − 05+	5.39*E* + 02 ± 2.75*E* − 01−	5.86*E* + 02 ± 1.52*E* + 02−	5.95*E* + 02 ± 7.69*E* + 00−	1.01*E* + 03 ± 1.87*E* + 00−	1.01*E* + 03 ± 1.41*E* + 00−	5.39*E* + 02 ± 1.62*E* − 02

−	12	16	11	15	12	8	/
+	7	5	7	3	9	12	/
=	2	0	3	3	0	1	/

“−,” “+,” and “=” denote that the performance of this algorithm is, respectively, worse than, better than, and similar to MDE according to the Wilcoxon signed-rank test at *α* = 0.05.

**Table 4:** Results obtained by the Multiple-Problem Wilcoxon test for twenty-one test functions at *D* = 10.

MDE versus	*R* ^+^	*R* ^−^	*p* value	At *α* = 0.05	At *α* = 0.1
CLPSO	169.0	41.0	0.016042	+	+
GL-25	184.0	47.0	0.016472	+	+
CMA-ES	192.0	18.0	0.001088	+	+
LBBO	141.0	90.0	0.366155	=	=
SFLSDE	147.0	63.0	0.112595	=	=
L-SHADE	136.5	73.5	0.232226	=	=

**Table 5:** Results obtained by the Multiple-Problem Wilcoxon test for twenty-one test functions at *D* = 30.

MDE versus	*R* ^+^	*R* ^−^	*p* value	At *α* = 0.05	At *α* = 0.1
CLPSO	200.0	31.0	0.003004	+	+
GL-25	198.5	32.5	0.003705	+	+
CMA-ES	177.0	54.0	0.031164	+	+
LBBO	160.5	70.5	0.113770	=	=
SFLSDE	184.0	47.0	0.016008	+	+
L-SHADE	116.5	114.5	0.95842	=	=

**Table 6:** Results obtained by the Multiple-Problem Wilcoxon test for twenty-one test functions at *D* = 50.

MDE versus	*R* ^+^	*R* ^−^	*p* value	At *α* = 0.05	At *α* = 0.1
CLPSO	155.5	75.5	0.157413	=	=
GL-25	179.5	51.5	0.024970	+	+
CMA-ES	136.0	95.0	0.465445	=	=
LBBO	175.5	55.5	0.035480	+	+
SFLSDE	138.0	93.0	0.424043	=	=
L-SHADE	94.0	116.0	1	=	=

**Table 7:** Average rankings of contraction criterion combinations by Friedman test at *D* = 10, *D* = 30, and *D* = 50.

*D* = 10		*D* = 30		*D* = 50	
Parameters	Ranking	Parameters	Ranking	Parameters	Ranking
*ρ*_1.0,1.0_	4.5714	*ρ*_1.0,1.0_	4.4286	*ρ*_1.0,1.0_	**4.2619**
*ρ*_1.0,2.0_	4.9286	*ρ*_1.0,2.0_	4.881	*ρ*_1.0,2.0_	4.4524
*ρ*_1.0,3.0_	4.9524	*ρ*_1.0,3.0_	4.381	*ρ*_1.0,3.0_	5.0714
*ρ*_2.0,1.0_	4.9762	*ρ*_2.0,1.0_	4.9286	*ρ*_2.0,1.0_	5
*ρ*_2.0,2.0_	**3.5714**	*ρ*_2.0,2.0_	**4.2381**	*ρ*_2.0,2.0_	5.0476
*ρ*_2.0,3.0_	6	*ρ*_2.0,3.0_	5.6667	*ρ*_2.0,3.0_	5.0238
*ρ*_3.0,1.0_	5.1429	*ρ*_3.0,1.0_	5.3571	*ρ*_3.0,1.0_	5.2381
*ρ*_3.0,2.0_	5.5714	*ρ*_3.0,2.0_	5.5952	*ρ*_3.0,2.0_	5.6905
*ρ*_3.0,3.0_	5.2857	*ρ*_3.0,3.0_	5.5238	*ρ*_3.0,3.0_	5.2143

**Table 8:** Average rankings of *C*_max_ by Friedman test at *D* = 10, *D* = 30, and *D* = 50.

*D* = 10		*D* = 30		*D* = 50	
Parameters	Ranking	Parameters	Ranking	Parameters	Ranking
*C*_max_ = 3	**3.4286**	*C*_max_ = 3	3.5238	*C*_max_ = 3	**3.6905**
*C*_max_ = 5	3.5476	*C*_max_ = 5	**3.3095**	*C*_max_ = 5	4.2381
*C*_max_ = 7	3.9762	*C*_max_ = 7	4.0476	*C*_max_ = 7	3.881
*C*_max_ = 9	3.881	*C*_max_ = 9	4.3333	*C*_max_ = 9	3.8095
*C*_max_ = 11	4.5238	*C*_max_ = 11	4.2381	*C*_max_ = 11	4.5238
*C*_max_ = 13	3.7143	*C*_max_ = 13	4.1429	*C*_max_ = 13	4.119
*C*_max_ = 15	4.9286	*C*_max_ = 15	4.4048	*C*_max_ = 15	3.7381

**Table 9:** Average rankings of *M* by Friedman test at *D* = 10, *D* = 30, and *D* = 50.

*D* = 10		*D* = 30		*D* = 50	
Parameters	Ranking	Parameters	Ranking	Parameters	Ranking
*M* = 30	**2.6429**	*M* = 30	**2.3095**	*M* = 30	3.1905
*M* = 60	2.7143	*M* = 60	2.6429	*M* = 60	2.7857
*M* = 90	2.8333	*M* = 90	2.9762	*M* = 90	**2.381**
*M* = 120	2.9762	*M* = 120	3.0238	*M* = 120	3.0952
*M* = 150	3.8333	*M* = 150	4.0476	*M* = 150	3.5476
